# SHIP-AGE: Frailty, renal function, and multi-component primary care in rural Mecklenburg-West Pomerania (MV-FIT)- study protocol

**DOI:** 10.1371/journal.pone.0324001

**Published:** 2025-06-09

**Authors:** Maik Gollasch, Yulia Komleva, Dmitry Tsvetkov, Felix Morof, Mladen V. Tzvetkov, Till Ittermann, Marcus Vollmer, Uta Zahn-Tesch, Holger Kock, Franziska Schuster, Neeltje van den Berg, Henry Völzke, Stefan Engeli, Lieven Kennes, Marwan Mannaa

**Affiliations:** 1 Department of Internal Medicine and Geriatrics, University Medicine Greifswald, Greifswald, Germany; 2 Department of Pharmacology, University Medicine Greifswald, Greifswald, Germany; 3 Institute for Community Medicine, Department SHIP/Clinical-Epidemiological Research, University Medicine Greifswald, Greifswald, Germany; 4 Institute of Bioinformatics, University Medicine Greifswald, Greifswald, Germany; 5 Strategic Research Management, University Medicine Greifswald, Greifswald, Germany; 6 Department of Clinical Pharmacology, University Medicine Greifswald, Greifswald, Germany; 7 Institute for Community Medicine, Section Healthcare Epidemiology and Community Health, University Medicine Greifswald, Greifswald, Germany; 8 Department of Economics and Business Administration, University of Applied Sciences Stralsund, Stralsund, Germany; PLOS: Public Library of Science, UNITED KINGDOM OF GREAT BRITAIN AND NORTHERN IRELAND

## Abstract

**Background.** Chronic kidney disease (CKD) is a leading risk factor for cardiovascular disease and all-cause mortality among older adults. Mecklenburg-West Pomerania has the highest CKD prevalence in Germany and Europe, however, its impact on frailty prevention strategies in primary care remains poorly understood. The SHIP-AGE/MV-FIT study aims to investigate the role of CKD in frailty incidence.

**Methods.** The SHIP-AGE/MV-FIT cohort is a prospective, longitudinal, population-based observational study targeting individuals ≥ 65 years with mGFR >30 mL/min. The cohort will consist of approximately 820 elderly participants who will be monitored over a three-year period. They will undergo a comprehensive, multi-factorial geriatric assessment, along with a structured monitoring and management program aimed at preventing frailty. The program incorporates evidence-based, multi-component care, including physical activity, medication review, nutritional optimization, and fall prevention strategies.

**Discussion.** SHIP-AGE/MV-FIT will clarify CKD’s role in frailty progression and identify mechanisms underlying frailty and pre-frailty. Additionally, the study aims to implement and evaluate multi-component healthcare strategies for frailty and fall prevention, assess patient adherence and quality of life, and explore elderly individuals’ experiences with primary care interventions. By integrating SHIP-AGE data with findings from the SHIP (Study of Health in Pomerania) cohorts in our region, this research will contribute to evidence-based strategies for maintaining health, independence, and well-being in aging populations, particularly in rural primary care settings, such as Mecklenburg-West Pomerania.

## Introduction

### 1. Relevance

The society is aging. Their health-care resources are major, and their medical problems are legion. Old patients often develop multiple chronic diseases and functional impairments. We treat these patients holistically; that is in addition to the specific illnesses, we take into account the individual family, social and life history, and functional reserve capacities of each individual patient. The focus is always on maintaining or regaining independence, quality of life and life perspectives for everyday life. Frailty in aging individuals, often accompanied by an increased risk of falls, leads to higher rates of hospital admissions, nursing home placements, and substantial economic costs, placing a significant burden on healthcare systems and society.

Frailty describes an aging-related syndrome of physiological decline, which is characterized by marked vulnerability to adverse health outcomes [1]. Frail older patients confer high risk for falls, which can lead to physical injury, psychological harm, or both [2]. Frailty is associated with reduced life expectancy, independence, quality of life and life perspectives, high risk of hospitalization and nursing home admissions of old and very old people. With aging renal function gets worse [3]. Mecklenburg-West Pomerania (MV) has the largest prevalence of chronic kidney disease (CKD) in Germany and Europe [4]. The prevalence of CKD is higher than the prevalence of type 2 diabetes (25%) and obesity (34%) among individuals aged 65 years or older S1 Fig [4, 5]. The impact of CKD on frailty is unknown, although even without renal disease, 66% of the elderly in MV has CKD KDIGO Stage 3 after 75 years merely by virtue of being old S1 Fig [4, 5]. CKD is common among older adults in Germany (BASE-II, SHIP studies), but awareness is low (1%) [4, 5]. Our study aims to clarify the burden of CKD on frailty in multi-component healthcare.

A 2016 review (DEGS1, ESTHE, KORA-Age, LUCAS cohorts) showed that the prevalence of physical frailty and pre-frailty in Germany (according to Fried phenotype [6]) were 3.1% and 38.3% in women and 2.5% and 35.9 % in men, respectively [7]. Falls occur at least once annually in 29% of community-dwelling adults 65 years or older — a rate of 0.7 falls per person-year [2]. With the demographic change in Germany, the proportion of the elderly in the population with fall injuries continues to rise. According to the 2016 report from the Federal Statistical Office, almost 6.8 million older people (65+) underwent surgery in Germany in 2014, with fall injuries being the third or second leading cause in men and women, respectively [8]. Multi-component intervention strategies (multi-component healthcare) are part of guideline-based care for reducing frailty and the risk of serious fall injuries of elderly persons [2]. However, the implementation of these intervention strategies in primary care is poor. We will introduce this intervention in primary care to reduce frailty in individuals aged 65 years or older in MV. Consequences should be less hospital admissions, less medication, better quality of life, longer life in their “own walls” and avoid acute illnesses. We will establish networks between hospital-based patient care and general practitioners (GPs), public, and private healthcare providers to bridge gaps between inpatient and outpatient primary care of old patients in rural MV. We expect that treatment and care across the in-hospital and primary care sector for elderly (Intersectoral Care Manangement, ICM) will stimulate new pathways of discharge management at the university hospital. Our strategy offers health care to the elderly keeping them as long as possible as active, non-frail and off-dialysis individuals that they can contribute to the society rather than being a “burden”.

### 2. Aim(s) and novelty

The Cardiovascular Health Study (4,637 participants) demonstrated that CKD is a leading risk factor for cardiovascular and all-cause mortality among elderly persons [9]. In Germany, end stage renal disease affects individuals who are on average 80 years old (QiN-Registry of KfH). The 5-year survival rate among all end stage renal disease hemodialysis patients is only 50% [10] (far less amongst the elderly) and most of these deaths are related to cardiovascular disease, making end stage renal disease a catastrophic risk factor [11]. Older frail dialysis patients often report accidental falls, but the role of renal dysfunction is unclear because of coexistence of factors such as polypharmacy, comorbidities and changes in volume status [12]. Because of the high prevalence, targeting frailty in conditions of reduced renal function is an important challenge which deserves clarification. Our central hypothesis is that reduced kidney function is a key risk factor and contributing driver of frailty and declining health status in the multi-factorial primary care of elderly individuals.

The objectives of this study are 1) to evaluate the impact of CKD on frailty among the elderly persons, and 2) to implement guideline-based, multi-factorial geriatric assessment, monitoring and managements (multi-component healthcare) in rural MV. We aim to achieve our objectives within the SHIP-AGE project through a prospective, longitudinal, observational study (SHIP-AGE/MV-FIT). We will implement guideline-based multi-component care to reduce frailty in all individuals aged 65 years or older with a particular focus on those with GFR 30-60 mL/min (KDIGO Stage 3). The prevalence of CKD is unprecisely determined by estimated glomerular filtration rate (eGFR) equations. We will directly measure GFR by the iohexol method and not rely solely on eGFR calculations [3]. Guideline-based, multi-factorial geriatric assessment, monitoring and managements (multi-component healthcare intervention) will be implemented with help of medical reports, geriatric health records and direct contact of the participants via tele-medicine (smartphone apps, email). Our strategy offers health care to the elderly to avoid the beginning of irreversible disabling processes, such as loss of muscle mass. Our intervention offers that disability can be avoided by detecting and treating frailty at an early stage and preventing potential decline related to malnutrition, lack of adequate physical activity, cognitive deterioration, incident falls or other problems. We emphasize on frailty in preventive aspects (both for clinicians and the general public) to reduce the healthcare burden in MV. Our study design will clarify the burden of CKD on frailty in multi-component healthcare. Our approach aims to contribute to improvement of ICM, which bridges gaps between geriatrics, GPs, specialists and community medicine at the interface between stationary and ambulatory medicine.

### 3. Evidence and objectives

The oldest definition of frailty, and one of the most widely used, is the phenotype developed by Fried *et al*. [6]. Frailty is defined as a clinical syndrome in which 3 or more of the following criteria are present: (1) weight loss, (2) slowness, (3) exhaustion, (4) weakness, and (5) low physical activity [6, 13] . Three large-scale studies addressed a possible role of reduced kidney function on frailty in the elderly. In all three studies, frail individuals were identified using the definition developed by Fried et al[6]. CKD stages were defined by KDIGO criteria (https://kdigo.org). 1) Dalrymple *et al*. investigated whether lower levels of kidney function were associated with prevalent or incident frailty in Cardiovascular Health Study (CHS, n=4,150; age ≥ 65 years) participants without stroke, Parkinson disease, prescribed medications for Alzheimer disease or depression, or severely impaired cognition [14]. The primary predictor was eGFR calculated using serum cystatin C (eGFRcys). Secondary analyses examined eGFR using serum creatinine (eGFRSCr). Outcomes were prevalent frailty and incident frailty at 4 years of follow-up. The authors found that lower eGFRcys was associated with a higher risk of prevalent and incident frailty whereas lower eGFRSCr was not. Nearly one fourth of older adults with an eGFRcys <45 ml/min per $1.73\;{\text{m}}^{2}$ (KDIGO <Stage 3a) had prevalent frailty. The findings were attenuated when the authors used the 2012 CKD-EPI cystatin C equation adjusted for demographic characteristics. However, the 2012 CKD-EPI cystatin C equation was not derived in an elderly cohort, and the age coefficient may not be optimal in the elderly. Thus, the authors were unable to demonstrate evidence for a role of reduced kidney function on prevalent or incident frailty. Similar results were observed in the Outcomes of Sleep Disorders of Older Men (MrOS Sleep) study cohort [15]. Together, the data highlight the importance of considering non-GFR determinants of kidney function, e.g. accelerated loss of lean muscle mass, which is thought to be an underlying process in frailty. 2) Ballew *et al*. investigated the cross-sectional association of frailty with kidney function. Kidney function was measured by several biomarkers (creatinine, cystatin C, and urine albumin) in The Atherosclerosis Risk in Communities (ARIC) Study cohort, which is biracial population-based cohort of men and women 66 years or older in the United States [13]. Among the 4,987 participants (men and women aged 66 years and older), 341 were classified as frail. Reduced eGFR (<60 mL/min/$1.73\;{\text{m}}^{2}$) was common in frail individuals, but the proportion with eGFRs <60 mL/min/$1.73\;{\text{m}}^{2}$ was much greater when kidney function was estimated using eGFRcys compared to eGFRScr (77% vs 45%). The authors concluded that cystatin C-based eGFRcys may be a much better marker of kidney function in frail individuals, with lower levels of eGFRcys independently associated with frailty. However, cystatin C level may be high in the setting of inflammation, which means that associations between frailty and eGFR based on cystatin C level may be driven by chronic inflammation rather than CKD itself. Often CKD is characterized by an inflammatory state, and higher inflammatory burden has been associated with frailty. 3) Guerville *et al*. investigated whether a fast decline on eGFR would be associated with incident frailty in community dwellers, aged ≥70 (n=833) in the Multidomain Alzheimer Preventive Trial (MAPT) [16]. The authors calculated eGFR using serum creatinine, age, sex in the eGFR CKD–EPI equation. Frailty occurred in 95 (11%) participants between 24 and 60 months. The authors concluded that people with fast eGFR decline are at risk for frailty incidence. However, since the difference between eGFR was determined at relatively short follow up intervals (24 months), this approach may have misclassified people with creatinine fluctuations instead of people with fast eGFR decline. Therefore, variations of serum creatinine may have accounted for variations of kidney function or muscle mass (possibly influenced by the multi-domain intervention), but were not addressed in this study [16].

**Fig 1:**
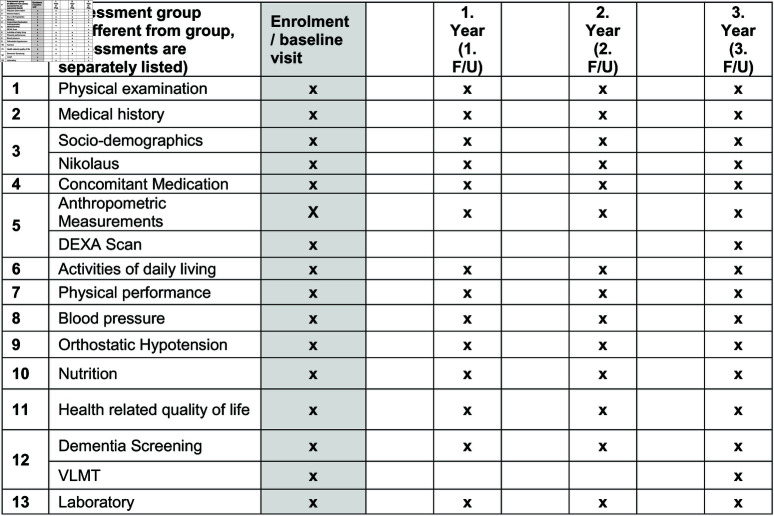
SPIRIT diagram: Assessments at baseline and follow up visits. F/U, follow-up visits.

**Fig 2:**
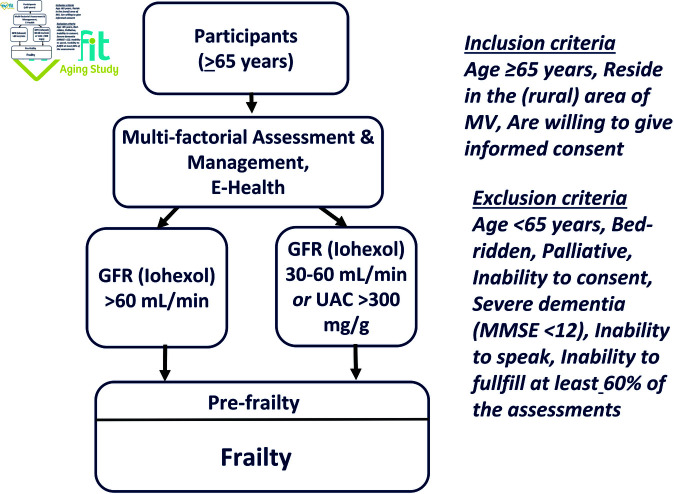
Protocol design. Inclusion and exclusion criteria.

**Fig 3:**
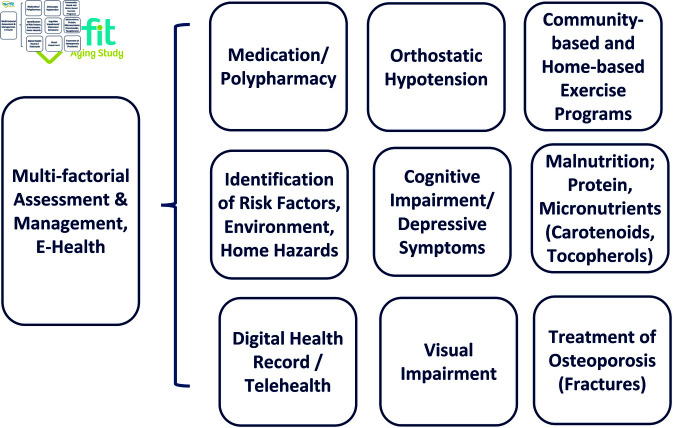
Multi-component intervention strategies. MMSE, Mini-Mental State Examination.

A 2018 systematic review concluded that available data do not support the superiority of one of the eGFR equations in measuring or predicting functional decline [17]. Estimating equations based on serum creatinine may overestimate GFR. Elevated cystatin C levels may indicate persistent chronic inflammation, which cannot be detected by elevated C reactive protein levels. There is only one study, which measured GFR (mGFR, ^125^I-iothalamate clearance) to study kidney function on self-reported frailty. The authors found an inverse association between both parameters. However, the data are difficult to interpret because frailty was not assessed by objective geriatric assessment tests [18]. There are no studies on the impact of reduced kidney function on frailty that involve long-term, population-based, epidemiological cohorts. Together, all the above studied relied on surrogate markers of kidney function rather than direct GFR measurements. Thus, we will perform a prospective, longitudinal, observational SHIP-AGE/MV-FIT study. A SPIRIT schedule and overview of the study design can be found in Fig 1. This study will implement direct measurements of GFR by the iohexol method (mGFR) to evaluate the impact of CKD in multi-component “real world” primary care strategies on lowering frailty among elderly persons in MV. We will study two groups of individuals; i.e. individuals aged 65 years or older with (i) directly measured GFR (mGFR) >60 mL/min and (ii) with mGFR 30-60 mL/min (KDIGO Stage 3) (Fig 2). All participants will undergo multi-component intervention strategies (multi-factorial geriatric assessment, monitoring and management systems (Fig 3) to clarify the impact of CKD on frailty in conditions of “best primary care” to reduce frailty and incident falls in the elderly [2, 19].

### Mecklenburg-Vorpommern Frailty Observation and Interventions Trial (MV-FIT)

Our SHIP-AGE/MV-FIT study is an observational study of individuals aged 65 years or older with mGFR >30 mL/min (n=820). All participants will receive multi-component healthcare, which includes multi-factorial geriatric assessment, monitoring and management systems (Fig 3) [2, 19, 20]. Renal function (mGFR) is measured by the iohexol method. Subjects will be stratified by mGFR. The incidence of frailty is observed over 36 months. High quality biobanking of longitudinally ascertained biosamples will allow biomarker research and basic science research into ageing syndromes while performing health care research and interventional studies. All patients will be encouraged to exercise [21]. We will recommend home-based exercise programs (e.g. Go4Life program) and/or group-based exercise programs for leg strength and balance (e.g. tai chi). Participants will be advised to participate in aerobic exercise training sessions three times weekly [22]. The algorithm to guide the selection of an exercise program is provided in Ganz *et al*. [2]. All prescribed and over-the-counter drugs will be reviewed [23]. Particular attention will be paid to medications that may cause sedation, confusion, or orthostatic hypotension [23]. All medications are reviewed with a focus on advising tapering or discontinuation of nephrotoxic or potentially inappropriate medication (e.g. PRISCUS guidelines). Patients who meet the criteria for dementia or depression will be evaluated or referred for reversible causes (e.g., for management of hypothyroidism). Ophthalmological examinations are recommended every 1 to 2 years [2]. Patients with balance deficits who wear multifocal lenses may also benefit from a referral for single-lens distance glasses to use when taking part in regular outdoor activities [24]. Participants who have orthostatic hypotension will be educated about rising slowly and not ambulating immediately after standing; potentially causative medications will be recommended for potentially causative medications will be recommended for discontinuing, and adequate hydration will be encouraged [2]. If poor nutritional status, i.e. malnutrition, is detected, we will recommend nutrition counseling or referral for a dietician [25]. Participants with previous vertebral or hip fracture after minimal trauma will be offered pharmacologic treatment for osteoporosis. Dual-Energy X-ray Absorptiometry (DexaScan) assessments will be reported to all participants and the primary health care providers, i.e. family doctors. As a complement to the SHIP-AGE/MV-FIT study, we will conduct a survey on the SHIP-START/SHIP-TREND cohorts in our region [26] to gain additional epidemiological insights into frailty. Participants (approximately 2,100 individuals currently aged ≥60 years) have been longitudinally followed for up to 24 years (SHIP-START) and 12 years (SHIP-TREND). This survey will assess frailty risk factors and disease progression, leveraging long-term cohort data to enhance our understanding of aging-related health outcomes in SHIP-AGE/MV-FIT.

### 4. Measurements

In the SHIP-AGE/MV-FIT study we will observe 820 participants over a period of 3 years. Informed consent will be obtained for participation in the study. Subjects who consent will be screened for eligibility and if the inclusion and none of the exclusion criteria are fulfilled, subjects will be enrolled. Data obtained from the screening, and enrollment visits must be supported in the patient’s source documentation, i.e. in the geriatric health record (eCRFs). Our follow-up (visits at 1st year and every 12 months) is designed to collect these data and to strengthen compliance to the individual interventions.

Screening Visits/Baseline: Adults aged 65 years or older will be enrolled if eligible. If eGFR (CKD-EPI) is known and >25 mL/min per $1.73\;{\text{m}}^{2}$, mGFR will be measured. Scheduled examination components and timeline by visits are shown in Fig 1.

Geriatric assessments and evaluations: Tables 1 and 2 show the assessments and evaluations. The GAITRite walkway system is the current gold standard in gait analysis. Leonardo mechanography, TUG, 5CRT are other physical measures.

**Table 1:** Frailty, renal function, and the effect of multi-component intervention strategies.

Design:	Prospective, longitudinal, observational study
All patient intervention:	Aerobic and resistance exercise and others (multi-factorial geriatric assessment monitoring and management systems)
Inclusion criteria:	Out-patients, aged ≥65 years, mGFR ≥30 mL/min per $1.73\;{\text{m}}^{2}$
Exclusion criteria:	Bedridden, palliative, inability to consent, severe dementia (MMSE<12), inability to speak, inability to fulfill at least 60% of the assessments
Primary outcome:	Frail vs. non-Frail
Secondary:	Frailty score transition (pre-frailty), mGFR transition, patient welfare, ability to live independently (off-dialysis), quality-of-life scales, burden of CKD on health status, cognitive decline, number of falls, admission to hospitals, referrals to nursing homes, all-cause mortality in primary care
Primary statistical method:	Multivariable logistic regression
Further statistical analysis:	Multivariable Cox regression, Chi-Square-Test, Propensity score matching
Assessments:	TUG, Tinetti, GAITRite, 5CRT, ADL, IADL, Barthel, Handgrip strength, medications, BMI, waist circumference, DexaScan, medical history (e.g. incontinence, falls), MNA, Nutrition protocols, Veggie Meter, social history, EQ-5D-5L, SF-12, MMSE, physical activity questionnaire, Leonardo Mechanography, VLMT, Money counting test, Watch test, GDS, Refractometry, Audiometry, Blood pressure, pulse-wave velocity, orthostatic (Tilt-table) evaluation, Finapres, Blood/Urine Metabonomics, DNA sampling
Main confounders:	mGFR (Iohexol), pre-existing frails, age, gender, BMI, multi-component compliance, comorbidities/medical history (diabetes, hypertension, etc.), Charlson comorbidity index, medications

Abbreviations: mGFR, measured glomerulation filtration rate; CKD, chronic kidney disease; TUG, timed up-and-go test; 5CRT, 5 chair rising test; ADL, Activities of Daily Living; IADL, Instrumental Activities of Daily Living; BMI, body mass index; MNA, Mini Nutritional Assessment; EQ-5D-5L, EuroQol 5-Dimension 5-Level Scale; SF-12, Short Form 12-Item Health Survey; MMSE, Mini-Mental State Examination; VLMT, Verbal Learning and Memory Test; GDS, Geriatric Depression Scale.

**Table 2:** Assessments in groups.

Assessment group	Data collection tool	Abbreviation
1. Physical examination	Weight loss	
	Exhaustion test	
	Leisure sport activity indices	
2. Medical history	History of chronic diseases	
	Number of falls, hospitalization and nursing homes	
	Well-being, comorbidities, incontinence	
3. Socio-demographics	Age	
	Ethnicity	
	Gender	
	Level of education	
	Marital status	
	Persons living with participants	
	Social history	
4. Smoking/alcohol	Alcohol intake	
	Binge drinking	
	Current smoking	
	Lifetime tobacco exposure	
5. Medication	Concomitant non-study medication	
6. Anthropometric Measurements	Body Mass Index	BMI
	Waist circumference	
	Body Composition	BIA
	Bone Density by Dual X-ray Absorptiometry	DEXA Scan
7. Activities of daily living	Activities of daily living, Barthel Index	ADL
	Instrumental activities of daily living	IADL
8. Physical performance	Handgrip strength	
	GAITRite^®^ examinations for temporal and spatial gait analyses	GAITRite
	Timed Up and Go mobility test (SmartVia)	TUG
	Performance-Oriented Mobility Assessment	Tinetti Test
	Chair rise test (Leonardo Mechanography^®^)	5CRT
	Leonardo Mechanograph^®^ Muscle Function and Performance	Leonardo
	Questionnaire-based surveys on frailty	
	Assessments of physical activity	
9. Blood pressure	Seated Blood Pressure/Pulse velocity	
10. Orthostatic Hypotension	Standing Blood Pressure/Tilt table/Finapres Nova^®^	Finapres
	Symptoms of orthostatic hypotension	
11. Nutrition	Mini Nutritional Assessment	MNA
	Nutrition protocols, Veggie Meter^®^	
12. Health related quality of life	Health-related quality of life	HRQL
	Geriatric depression scale	GDS
	Generic health status	EQ-5D-5L
	Health related quality of life questionnaire	QOL
	12-Item Short Form survey	SF-12
13. Dementia Screening	Mini Mental State Examination	MMSE
	Verbal learning and memory test	VLMT
	Money counting test	
	Watch test	
14. Laboratory	Blood and Urine Collection	

Schedule of Follow-Up Visits and Procedures by Visit: Post-enrolment follow-up visit schedules for data collection do not differ between both mGFR groups. All participants will have post-enrolment visits every 12 months thereafter (Fig 1). The trial is testing the effectiveness of a treatment strategy question regarding the burden of CKD on frailty and all its consequences on health status in the elderly and not testing specific treatments. All subjects will undergo multi-component digital intervention strategies, which include multi-factorial geriatric assessment, monitoring and management systems intervention and eHealth (Fig 3). The intervention protocol is strict in medical advice and interventional details. However, it is flexible in terms of choice of individual treatments (i.e. acceptance to implement recommendations, environmental assessments and modification, extent and degree of aerobic and/or resistance exercise, single-lens distance glasses for outdoor activities etc.). Multi-component compliance will be monitored and studied in regression model to analyse the implementation of the individual interventions. Our follow-up (visits at 1st year and at least every 12 months) is designed to collect these data and to strengthen compliance to the individual interventions. We will follow the participants with geriatric health records to document the objectives.

## Materials and methods

### General design

SHIP-AGE/MV-FIT is a prospective, longitudinal, population-based observational study, designed in accordance with STROBE guidelines. Recruitment period of the study from 2024-04-03 to 2025-09-30.

### Research ethics approval

This study was approved by the University Medicine Greifswald Ethics Review Board on 30th of June 2023 with identification number: BB 161/22. The research protocol has been preregistered at clinicaltrials.gov under the record identifier NCT05962203.

### Study setting

Adults aged 65 years or older in Mecklenburg-West Pomerania (MV) will be included. All sexes are eligible for participation. Baseline and follow-up examinations will take place at the University Study Center at the Hospital Wolgast. The recruitment is monitored very closely so that, if necessary, adaptations can be made in order to avoid large delays. These objectives may be realized through the inclusion of additional study sites, such as the University Medical Center in Rostock—the other university in MV with which we maintain strong collaborations.

### Eligibility criteria

Inclusion/exclusion criteria were made as simple as possible to recruit a largely heterogeneous (“real world”) elderly population.

- Inclusion Criteria: Age 65 years or older, mGFR >30 mL/min per $1.73\;{\text{m}}^{2}$, being able to understand and give written informed consent.
- Exclusion Criteria: Bedridden, palliative, inability to consent, severe dementia, inability to speak, lack of compliance (paracusis, inability to fulfill at least 60% of the assessments).

Recruitment is promoted through various regional media and communication channels. These include advertisements in local newspapers, publications in municipal bulletins, flyers, and information on websites and digital platforms. Additionally, local networks and community structures are utilized to reach interested older adults and encourage broad participation. We will establish networks with ambulatory practices and stakeholders to recruit and follow the patients. In terms of CKD patients, we will contact local nephrologists in practice. All sites are located in MV.

Participants will be stratified by mGFR: into (a) individuals with mGFR, 30-60 mL/min per 1.73 m2 or urine albumin/creatinine ratio (UAC) ≥300 mg/g and (b) individuals with mGFR, >60 mL/min per $1.73\;{\text{m}}^{2}$ and urine albumin/creatinine ratio (UAC) <300 mg/g. Approximately 328 participants (40%) are expected to have mGFR, 30-60 mL/min per $1.73\;{\text{m}}^{2}$ and 492 participants (60%) are expected to have mGFR, >60 ml/min/$1.73\;{\text{m}}^{2}$ (Fig 2). The planned minimum follow-up duration for participants is three years, while the maximum follow-up duration is approximately six years.

### Subjects information and consent

Interested individuals will receive written information a few days before their enrollment visit and will be informed in person upon arrival at the study center. Both written and oral consent will be required for participation.

### Study timeline

Study start: 2023-09-01; Study completion (estimated): 2030-09-30

### Study outcomes

The primary outcome will be frailty at 36 months post subject inclusion (i.e. Frail vs. non-Frail). Secondary endpoints are: Frailty score transition (pre-frailty), mGFR transition, patient welfare, ability to live independently, quality-of-life scales, burden of CKD on health status, cognitive decline, number of falls, admission to hospitals, referrals to nursing homes, all-cause mortality in primary care. Secondary endpoints will be determined using a broad spectrum of standard geriatric and research assessments as shown in Table 1 (for Abbreviations: see Table 2). Identification of novel parameters may be helpful for early detection of patients at risk for falls.

### Sample size

We performed a sample size calculation for the SHIP-AGE/MV-FIT cohort. A 3-year prevalence of frailty of 20% is expected in individuals with mGFR 30-60 mL/min per $1.73\;{\text{m}}^{2}$ and a prevalence of 12.5% in individuals with mGFR >60 ml/min/1.73 m2. The effect size of the GFR group on frailty is expressed by the odds ratio (OR) between mGFR-group 30-60 mL/min per $1.73\;{\text{m}}^{2}$ compared to the reference group mGFR >60 mL/min per $1.73\;{\text{m}}^{2}$. In multivariable adjusted models with frailty as independent variable, OR per 10 mL/min/$1.73\;{\text{m}}^{2}$ decrease of mGFR was reported to be 1.2 (Cardiovascular Heart Study) [14] and 1.4 (Modification of Diet in Renal Disease study) [18]. An OR of 1.83 was reported in multivariable logistic regression analysis when comparing mGFR 30-44 to ≥45 mL/min per $1.73\;{\text{m}}^{2}$. The target sample size was computed with G*Power by expecting a multivariable adjusted effect size of OR=1.75 in logistic regression analysis with 3-year frailty as the binary response variable. Statistical power was set to 80%, type I error rate was set to 5% and group imbalance is expected (40% will have mGFR, 30-60 mL/min per $1.73\;{\text{m}}^{2}$). Under these conditions one-sided testing led to the required sample size of 616 individuals to be analysed. To further adjust for drop-outs (lost-to-follow-up, deaths, no longer willing or capable to participate in the study), the sample size was corrected for a 25% drop-out rate justifying that 820 volunteers will be enrolled at baseline. The overall goal for recruitment is 820 participants, although the final enrolment number may be between 700 and 1,000.

### Data management

Data and documents are documented on the eHealth platform of the University Medicine Greifswald. Data reports are regularly extracted from the eHealth platform to check completeness and plausibility of the data (quality assurance). Data collections will be accompanied by a continuous data monitoring with standardized reporting.

### Status of study

As of March 5, 2025, more than 2,000 eligible individuals have been registered, with 505 participants enrolled in the study.

### Statistical methods

Evaluation of the impact of CKD on frailty among elderly persons The primary outcome Frail vs. non-Frail and events observed in the follow-up period will be analyzed using multivariable logistic regression. Furthermore, in case of proportional hazards, multivariable Cox regression analysis will be conducted to study the time to frail, which usually has a higher statistical power than logistic regression. Main exposure variables that enter both models are mGFR at baseline, pre-existing frails, frailty-index, sex, BMI, age, comorbidities/medical history, medication, geriatric depression score, and quality of life (see assessments). In univariable analysis all possible predictor variable will be checked in relation to the response variable whether a transformation of axis (e.g. log-transformation), spline-usage, or (re-)categorization seems appropriate and medically plausible for the use in multivariable models. In case of mGFR, we include baseline measurements, gender-specific quantile probabilities, and absolute and relative mGFR changes if available. Interaction terms will be used in model equations based on prior knowledge (evident interactions) and other (currently unknown) interaction effects will be tested for a significant increase in the model performance. Event variables (obligation to dialysis, hospital re-admission, referrals to nursing homes, all-cause mortality, etc) will be analyzed with penalized multivariable logistic regression. In order to create complete cases, we make use of imputed datasets (e.g. by Multiple imputation using Fully Conditional Specification or by Random Forest imputation). The model size will be controlled by setting regularization parameters (Elastic net or LASSO) and repeated internal cross-validation will be used to avoid overfitting and to generate more robust estimates that can result in a very generalizable model. Secondary endpoints such as the patient-individual changes in mGFR and in the frailty score will be analyzed in linear mixed effects models. Implementation of multi-component healthcare will be monitored by usage of the regional digital health records. If necessary, the results of formative process analysis are used to adapt processes or the documentation.

### Monitoring

Oversight and supervision of the trial will be documented by a study monitor. The execution of the trial will be in line with the study protocol and its amendments as well as Declaration of Helsinki in its current version and effective legal regulations. The study monitor is authorized to inspect the source data (e.g. data from laboratory diagnostics, medical records) for comparison with the CRF. He has to act with regard to the European General Data Protection Regulation. Quality assurance / monitoring will comprise monitoring and audits (preparation and visiting of trial sites), site management, study protocol and CRF development and submission to authorities.

### Protocol amendments

New approval will be sought out for any protocol amendments (e.g., eligibility criteria, allocation, intervention protocol).

### Consent or assent

Informed consent from each participating subject will be signed. All study specific documents and prove of qualified personnel undergo an assessment by the ethics committee. The study is conducted in accordance with the Declaration of Helsinki in its current version, and all applicable subject privacy requirements. With subjects who have cognitive impairments, the person assigned as durable power of attorney is initially present during the informed consent process.

### Dissemination policy

The trial results will be communicated to participants, healthcare professionals and the public via publication in medical journals. There are no planned publication restrictions. Findings will also be presented at conferences and scientific meetings. The research data will be made available taking into account data protection law and ethic, guidelines and recommendations for other researchers. Educational lectures will be given.

## Discussion

The SHIP-Age/MV-FIT study is designed to clarify the role of CKD on frailty and to identify underlying mechanisms of frailty and pre-frailty. A major strength of our study is its comprehensive approach, integrating multi-component healthcare strategies aimed at frailty and fall prevention, patient adherence, and quality of life assessment. By analyzing SHIP-AGE/MV-FIT data, this research will contribute to evidence-based interventions for preserving health, independence, and well-being in aging populations, particularly in rural primary care settings.

A key focus is on renal function, nephrotoxicity (particularly from pain medications), highlighting the impact of CKD on frailty and health deterioration. Our study will actively engage with general practitioners, public and private healthcare providers, and local social network systems, including fitness clubs, running groups, community organizations, and other social meeting points for the elderly. This broad community integration is essential, particularly given the limited number of geriatric centers in the region, making effective information dissemination crucial for the success of the study.

Another major strength of SHIP-Age/MV-FIT is the access to comprehensive medical records, allowing us to incorporate relevant health conditions such as stroke, heart failure, cancer, and gastrointestinal disorders, further refining our understanding of frailty risk factors. Furthermore, analyzing the SHIP-AGE/MV-FIT cohorts alongside the SHIP cohorts will allow for a comprehensive evaluation of frailty risk factors and disease progression. Propensity score matching will enable comparisons between participants with similar baseline characteristics, providing a clearer understanding of the impact of chronic kidney disease (CKD) and multi-component healthcare interventions on frailty and fall prevention. Integrating long-term data from SHIP-START and SHIP-TREND will further refine insights into aging trajectories, facilitating the identification of modifiable risk factors and personalized interventions. The SHIP cohorts serve as a parallel Pomeranian control group, enhancing the study’s value through an established research infrastructure.

By implementing direct GFR measurements in clinical routine and introducing novel technologies such as GaitRite, Finapres Nova, and Leonardo mechanography, we aim to improve the detection and characterization of pre-frailty syndromes. Conducting this study at a University Medical Center (Greifswald/Wolgast) will further promote the dissemination of findings across Mecklenburg-West Pomerania, ensuring broader clinical impact. Additionally, if successful, the strategies and structures implemented could serve as a model for other rural regions, helping to address demographic challenges in an aging society and improve health outcomes and primary care for the elderly.

Given the interconnection between aging, CKD, and frailty, our findings may indicate that early interventions targeting CKD could be beneficial not only for the general geriatric population but especially for CKD patients, who face a significantly higher risk of frailty. As CKD reaches epidemic levels in the elderly, targeted strategies for early detection, treatment, and comprehensive geriatric assessment will be essential. A personalized approach that clearly delineates individual deficits will allow for more precise, tailored therapeutic interventions, ultimately improving patient outcomes and quality of life in the aging population.

## Supporting information

S1 Fig Prevalence of chronic kidney disease (CKD) in Mecklenburg-West Pomerania (MV). Study of Health in Pomerania (SHIP-START-0, SHIP-TREND-0). eGFR, estimated glomerular filtration rate; yrs, years; UACR, urine albumin/creatinine ratio. CKD-EPI Creatinine Equation.
(PDF)
