# Supplementary material for: SHIP-AGE: Frailty, renal function, and multi-component primary care in rural Mecklenburg-West Pomerania (MV-FIT)- study protocol
Source: PLoS One. 2025 Jun 9;20(6):e0324001. doi: 10.1371/journal.pone.0324001 (PMC12148103; doi:10.1371/journal.pone.0324001)
Supplement: S1 Fig — Study of Health in Pomerania (SHIP-START-0, SHIP-TREND-0). eGFR, estimated glomerular filtration rate; yrs, years; UACR, urine albumin/creatinine ratio. CKD-EPI Creatinine Equation. (PDF) [file pone.0324001.s001.pdf]

Prevalence

53.7%[#] **[CKD-EPI]** equation

< **80.6% Hypertension**

> **25.4% Diabetes**
> **33.7%* Obesity**

CKD≥G3a

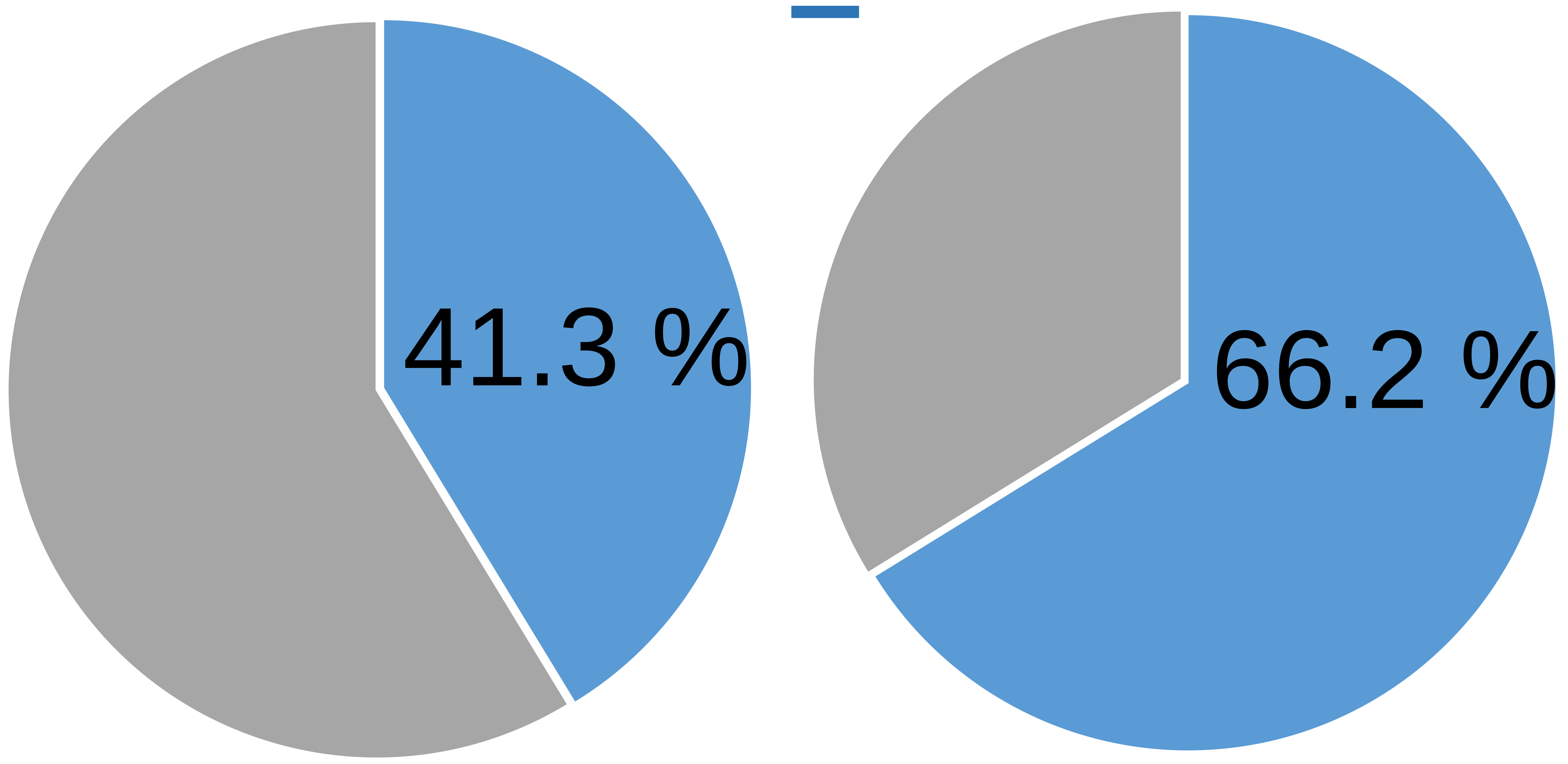

65-74 yrs   75-84 yrs

N= 984 subjects;
Ages 65+

No CKD: eGFR ≥60 mL/min/1.73 m^2, UACR<300 mg/g

Figure S1.
